# Mental health, behavioural problems and treatment seeking among students commencing university in Northern Ireland

**DOI:** 10.1371/journal.pone.0188785

**Published:** 2017-12-13

**Authors:** Margaret McLafferty, Coral R. Lapsley, Edel Ennis, Cherie Armour, Sam Murphy, Brendan P. Bunting, Anthony J. Bjourson, Elaine K. Murray, Siobhan M. O'Neill

**Affiliations:** 1 School of Psychology, Ulster University, Magee Campus, Derry/Londonderry, United Kingdom; 2 Northern Ireland Centre for Stratified Medicine, Biomedical Sciences Research Institute Ulster University, C-TRIC, Altnagelvin Hospital, Derry/Londonderry, United Kingdom; 3 School of Psychology, Ulster University, Coleraine Campus, Coleraine, United Kingdom; Shinshu University School of Medicine, JAPAN

## Abstract

Mental health and behavioural problems are common among students commencing university. University life can be stressful and problems often exacerbate during their course of study, while others develop disorders for the first time. The WHO World Mental Health Surveys International College Student Project aims to conduct longitudinal research to examine and monitor student mental health and wellbeing. The Ulster University Student Wellbeing study, which commenced in September 2015 in Northern Ireland (NI), was conducted as part of this initiative (wave 1, *n* = 739), using the WMH-CIDI to examine psychopathology. Baseline prevalence rates of lifetime and 12-month mental health and substance disorders, ADHD and suicidality were high, with more than half of new undergraduate students reporting any lifetime disorder. Co-morbidity was common with 19.1% of students experiencing three or more disorders. Logistic regression models revealed that females, those over 21, non-heterosexual students, and those from a lower SES background were more likely to have a range of mental health and behavioural problems. Overall, 10% of new entry students received treatment for emotional problems in the previous year. However, 22.3% of students with problems said they would not seek help. The study provides important information for universities, policy makers and practice, on mental health and wellbeing in young people generally but particularly for students commencing university. The findings will assist in the development and implementation of protection and prevention strategies in the university setting and beyond.

## Introduction

Many mental health problems emerge before the age of 18 with the period from 18 to 25 being a particularly susceptible developmental time in a person’s life [1]. For many young adults, this may coincide with their time at university. Starting university is a key life transition, which can be extremely stressful for some people. For a number of students, pre-existing mental health problems may be aggravated during this transition. These problems may persist or indeed worsen during their course of study and can have an impact on academic performance [2–4]. For others, however, the stress of university life and adapting to a new social environment can trigger psychopathology [5,6].

Recent research has revealed a high and increasing prevalence of psychopathology among students in higher education [7]. Indeed, the issue is becoming a growing concern globally [8–10]. For example, in 2014, a large-scale study reported that 32.6% of American students said that they were so depressed they found it difficult to function, 54% reported overwhelming anxiety and 8.1% had seriously considered suicide [11]. In 2015, depression rates had increased to 36.1%, anxiety to 58.6%, and seriously considering suicide rose to 10.3% [7]. In relation to clinical estimates, the Healthy Minds study of over 14,000 American college students reported that 17.3% met the criteria for depression, 7% for generalised anxiety disorders, 4.1% for panic disorders, 6.3% for suicide ideation and 15.3% for self-harm [12].

High prevalence rates of mental health problems and suicidality have also been found among university students in the UK. For example, when compared to their pre-entry levels, undergraduate students reported increased levels of anxiety during their first year [6,13], and poorer psychological wellbeing throughout their time at university [6]. Another study [14] reported that nearly a quarter of first year students had clinical levels of psychological distress. Furthermore, it has suggested that the increases in psychological problems may be related to widening participation in UK universities, with 17.3% of students having mental health problems, similar to rates found in the general population [15].

In addition to adapting to university life, many students are also adapting to their transition into adulthood. It has been suggested that the first few months at university are particularly challenging and stressful due to numerous psychosocial adjustments [16]. Some students may be living away from their family and friends for the first time. Others may be coming directly from schools, which were very structured, and they may find it difficult to adapt to a less structured academic setting [17]. Many older students often have the added responsibility of caring for family or work commitments, which can lead to added stress.

A large-scale study conducted in an Australian university reported that females, those aged 25–34, students with a low income and non-heterosexual students had the highest rates of mental health problems [18]. Additional studies corroborate that females have significantly higher rates of mental health problems [12,19,20]. Students who struggle financially also have significantly higher rates of mental health problems [5,21] than those who do not report financial concerns. Indeed, research has revealed that deprivation has a very negative impact on mental health in NI [22]. Furthermore, high rates of mental illnesses are especially common in those who identify as bi-sexual or homosexual [23].

Moreover, while mental health problems are highly prevalent and persistent, research has shown that many students who meet the criteria for a disorder do not receive treatment [2]. For example, a study found that only 36% of students in the US who had a mental health problem received treatment in the previous 12 months [24]. The uptake of treatment was even lower in a recent cross-national study which examined findings from 21 countries, with only 16.4% of students receiving treatment for their mental health problems in the previous year [4].

Significant gender differences in help seeking have also been found, with 11% of female students looking for help in comparison to 6% of males [25]. A global survey found that while males made up 43.8% of the student body, they only comprised of 33.9% of clients who presented to college counselling centres [26], suggesting that males tend not to seek help for mental health problems. While females generally have higher rates of mood and anxiety disorders [26] this only partially accounts for the gender difference found in help seeking.

While some studies [15,27] reported comparable rates of mental health disorders between the general population and university students, other studies have found significantly higher prevalence rates among students [21,28]. For instance, a study conducted in an Australian university reported elevated prevalence rates in the student population when compared with the general population, with a quarter of students experiencing very high levels of psychological problems [29]. The first onset of suicidal thoughts and behaviours have also been found to be higher among college students in comparison to the general population [3].

However, cross-national research carried out by the WHO World Mental Health Survey Initiative revealed that 20.3% of students had a 12-month mental health disorder in comparison to 21.4% of non-students in the population. Conversely, the highest level of 12-month mental health problems was found in those who had commenced university but failed to graduate, with 25% of those who dropped out reporting psychological problems [4]. Additionally, those with mental health problems may be less likely to get a place in university, which may partially account for the slightly higher rate of psychopathology found in some studies for non-students [4].

Studies corroborate that mental health problems can impact severely on a student’s life [30]. Indeed, mental health problems considerably disrupt learning ability [10], with psychopathology, particularly anxiety and depression, being associated with lower grades [31]. In addition, students who had lifetime suicide plans and attempts when entering university obtained significantly lower grades [3], as did those who engaged in non-suicidal self-injury [32]. Issues with attention and concentration can also impact on grades in addition to mental wellbeing. For example, ADHD is often co-morbid with a range of mental health disorders [33]. Moreover, research has found that of those with DSM IV/CIDI mental health disorders in the previous 12 months, 83.1% of disorders commenced before students started college and that pre-matriculation onset was associated with higher attrition rates and lower university entry rates [4].

It is important therefore to establish baseline prevalence rates of disorders and to understand the socio-demographic predictors of mental health and behavioural problems when students first enter universities. This point of entry information may be very beneficial for universities, helping them to provide adequate support for students and addressing problems early, minimising risk and improving grades and retention rates. A report examining the mental health of students in higher education recommends the use of longitudinal studies to gain greater insight into psychopathology in the student body [9]. Research such as that carried out by the WHO World Mental Health Surveys International College Student Project (WMH-ICS) will gather important information about the wellbeing of the student population. Conducted as part of this initiative, the Ulster University Student Wellbeing Study aims to examine and monitor student health and wellbeing during their time at university.

The main aims of the current study are to provide baseline prevalence rates of mental health problems, ADHD and suicidal behaviour among first year undergraduate students, who have newly registered at Ulster University, NI. The study also examines gender variations and age of onset in reported lifetime and 12-month disorders. Socio-demographic correlates of mental health disorders will be explored using logistic regression. Help seeking behaviour and the use of medication or counselling for mental health problems will subsequently be examined.

## Method

### Design

In September 2015, the Ulster University Student Wellbeing study (UUSWS) commenced on four campuses across Northern Ireland (Belfast, Coleraine, Derry/Londonderry and Jordanstown). The UUSWS study is being conducted as part of the WHO World Mental Health International College Student Project (WMH-ICS).An observational, longitidunal cohort study design is used for all studies. Prospective studies, such as this, can be very benefical in that recall issues are minimised, sequences or patterns of events can be established and causal relationships may be inferred. Ethical approval was obtained for the Northern Ireland study from the Ulster University Research Ethics Committee (REC/15/0004).

### Sample

Overall, 4,365 first year undergraduates registered at Ulster University in the year 2015–2016. A week prior to registration an email was circulated to all new first year undergraduate students due to register at the university, with a detailed participant information sheet attached outlining the aims of the study and methodology to be employed. Students were asked to consider participating in the study. Trained researchers recruited students on the various campuses following registration. A total of 1,646 participants provided informed written consent to take part in the study. Saliva/DNA samples (4ml) were collected from each participant using the Oragene-500 (DNA Genotek) self-collection kits according to the manufacturer’s instructions and stored for genetic analysis. Each student was provided with a card containing their unique ID number and a link to the survey. The survey instrument was administered on-line using Qualtrics software. The link to the survey was also emailed to each participant. The survey was fully completed by 739 of the participants. The completed response rate was 16.95% in relation to the total number of first year students registered. While other students partially completed the survey only those who responded to all questions are included in the analyses for the current study (*N* = 739), of which 462 were female, 274 were male, and three students identified as other (1 transgender male to female, 1 transgender female to male and 1 non-binary). The average age of participants was 21(*M* = 20.69, *SD* = 5.313), with an age range of 18 to 49. All participants were residents of the United Kingdom (UK) or the Republic of Ireland (ROI). International students, those repeating first year and students under the age of 18 were excluded from the study.

### Diagnostic assessment

The WMH-ICS surveys use questions adapted from the WMH Composite International Diagnostic Interview (CIDI), version 3.0, [34] to explore the prevalence of mental health problemsin accordance with ICD and DSM criteria. Good concordance has been found between the WMH-CIDI and clinical assessments [35]. The instrument includes screening sections for depression, bi-polar disorder, anxiety, panic attacks or panic disorder and other serious emotional problems. The Alcohol Use Disorders Identification Test (AUDIT), developed by the WHO [36], was used to screen for probable alcohol dependence. Drug abuse and dependence was measured using The Alcohol, Smoking and Substance Involvement Screening Test (ASSIST), which has been found to be a reliable [37] and valid screening test for drug use [38]. ADHD was assessed using the WMH-CIDI screener for Attention-Deficit/Hyperactivity Disorder, with questions focusing on difficulties with attention and concentration in the previous 6 months. Suicidal thoughts, plans and attempts were assessed using items from the Self-Injurious Thoughts and Behaviour Interview, the SITBI [39], an instrument with strong psychometric properties. At the end of the survey a list of useful resources were provided for participants with links to information, help and support. If students were deemed to be at risk of suicide, due to their responses to certain questions, such as if they attempted or planned suicide in the previous year, an alert was triggered. Carecall, the university counselling provider, contacted these students by phone to establish if they required help and provided information on support available.

### Help seeking and treatment for emotional problems

Questions from the WMH-CIDI services section were adapted and used to assess help seeking behaviour and receipt of treatment, including medication or counselling for emotional problems. For example, students were asked how likely they would be to go to the student counselling provider or someone else, such as a doctor or mental health professional if they developed a serious emotional problem in the coming year. Students were also asked if they had received either psychological counselling or medication for an emotional problem, how old they were when they first received treatment, if they were still in treatment and how old they were the most recent time they received treatment.

### Data analysis

Weights were created using the gender and age characteristics of the first year student population at Ulster University, subsequently these were applied to analyses to ensure that the study results wererepresentative of the total student population. Lifetime and 12-month prevalence rates for a range of mental health and substance disorders, ADHD and suicidality were calculated. Any disorder included any lifetime mental health disorder or suicidality or substance disorder or 6-month ADHD. Chi-square tests for independence examined gender differences in prevalence rates. The age of onset was examined, with independent samples t-tests conducted to determine gender variations. Differences in help seeking and use of medication or counselling in the previous year between males and females and those with and without disorders were examined using chi-square tests for independence. Logistic regression analyses were used to explore relationships between mental health problems and gender, age (21 and over, under 21), sexuality (heterosexual, non-heterosexual), current finances (poor, enough, comfortable, well-to-do) and where the person grew up (large city, suburbs, small city, town or village, rural setting). All analyses were conducted using SPSS (version 23).

## Results

Lifetime prevalence rates of mental health disorders, substance disorders, suicidality and 6-month ADHD are presented in Table 1. The highest prevalence rates were found for suicidality (31%), major depressive episode (24.2%) and generalised anxiety disorder (22.6%). Chi-square tests for independence revealed significant gender differences in reported rates. Females were significantly more likely to experience mood and anxiety problems as well as suicidality. Males had significantly higher rates of drug abuse and dependence but numbers were very low (4.8%). The overall prevalence rate for experiencing any lifetime disorders was 53.2%, with females experiencing significantly higher rates than males. Overall, 19.1% of students including the three people who identified themselves as either transgender or non-binary experienced 3 or more disorders.

**Table 1:** Lifetime prevalence of mental health and substance disorders, suicidality and 6-month ADHD.

	Total	(739)	Male	(274)	Female	(462)	Other (3)	
Disorders	*n*	%	*n*	%	*n*	%	*n*	χ ^2^
Mood—MDE	186	24.2	55	19.1	128	27.7	3	6.756**
Anxiety—GAD	173	22.6	49	17.8	121	26.0	3	6.312*
Panic Disorder	49	6.3	14	4.9	35	7.5	0	1.683
Broad Mania	27	3.5	7	2.5	19	4.1	1	1.567
Alcohol dep	75	10.2	31	11.1	44	9.5	0	.329
Drug abuse/dep	23	3.1	1.5	4.8	8	1.7	0	9.102**
Suicidality	237	31.0	68	24.3	166	35.9	3	10.329**
6-month ADHD	156	20.8	52	18.7	101	21.9	3	.339
Any disorder	400	53.2	135	48.5	262	56.6	3	4.215*
One disorder	172	23.2	63	23.0	109	23.5	0	
Two disorders	80	10.9	31	11.4	49	10.6	0	
Three or more	148	19.1	41	14.1	104	22.5	3	

Note: n = raw unweighted values, % weighted values, other = transgender or non-binary, MDE = major depressive episode, GAD = generalised anxiety disorder, Suicidality includes lifetime suicide thoughts, plans or attempts, ADHD = Attention deficit hyperactivity disorder, Any disorder = Any lifetime mental health disorders or suicidality or substance problems or 6 month ADHD, *X*^*2*^tests show significant gender differences in prevalence rates.

*p < .05

**p < .01

***p < .001.

Table 2 reports 12-month prevalence rates for a range of mental health disorders, substance dependence or abuse, suicidality and 6-month ADHD. The number of people who met the criteria for any 12-month disorders were high at 47.5%, although rates were lower than lifetime prevalence rates. Greater variations between lifetime and 12-month prevalence rates was found for suicidality. Gender differences were found in 12-month prevalence rates for mood and anxiety disorders, with females reporting significantly higher rates than males. Significant differences were also revealed for drug abuse or dependence, with males having higher rates (3.8%) than females (0.2%). Again, it should be noted that numbers were very low. In contrast to lifetime prevalence rates for suicidality, no significant gender difference was found in 12-month rates. Additionally, no significant gender variations were found in prevalence rates for any disorders reported in the previous year. The overall rate of any disorders reported in the previous 12-months remained high however at 47.5%, with 15.3% experiencing 3 or more disorders.

**Table 2:** 12-month prevalence of mental health and substance disorders, suicidality and 6-month ADHD.

	Total	(739)	Male	(274)	Female	(462)	Other (3)	
Disorders	*n*	%	*n*	%	*n*	%	*n*	χ ^2^
Mood—MDE	163	21.4	47	16.8	113	24.5	3	5.310*
Anxiety—GAD	167	21.9	47	17.2	117	25.2	3	6.388*
Panic Disorder	40	5.2	11	4.1	29	6.3	0	1.713
Broad Mania	20	2.6	6	2.2	13	2.8	1	.234
Alcohol dep	68	9.3	29	10.3	39	8.5	0	1.584
Drug abuse/dep	12	1.8	11	3.8	1	0.2	0	17.070*
Suicidality	137	18.5	44	16.3	90	19.6	3	1.435
6-month ADHD	156	20.8	52	18.7	101	21.9	3	.339
Any disorder	385	47.5	126	45.5	225	48.7	3	.539
One disorder	161	21.9	64	23.4	97	21.0	0	
Two disorders	75	10.2	31	11.1	44	9.6	0	
Three or more	118	15.3	31	11.1	84	18.2	3	

Note: n = raw unweighted values, % weighted values, other = transgender or non-binary, MDE = major depressive episode, GAD = generalised anxiety disorder, Suicidality includes 12-month suicide thoughts, plans or attempts, ADHD = Attention deficit hyperactivity disorder, Any disorder = Any 12-month mental health disorders or suicidality or substance problems or 6 month ADHD, *X*^*2*^tests show significant gender differences in prevalence rates.

*p < .05

**p < .01

***p < .001.

The age of onset for a range of mental health and substance disorders, suicidality and ADHD are presented in Table 3. The average age of onset for most problems was between 14 and 17. However the average age of onset for 6-month ADHD was lower (*M* = 11.5, *SD* = 5.36). Significant gender differences in average age on onset for ADHD were also revealed, with males having onset at a younger age (*M* = 10.09, *SD* = 4.77) than females [*M* = 12.37, *SD* = 5.60; *t*(135) = -2.484, *p* = .014]. Some problems started as early as 4, with absolute values of between 8 and 41.

**Table 3:** Age of onset of mental health disorders, substance disorders, suicidality and 6-month ADHD.

	Total			Male			Female		
Disorders	range	Mean	SD	range	Mean	SD	range	Mean	SD
Mood–MDE	4–45	15.5	4.58	11–45	16.5	4.74	4–40	15.0	4.46
Anxiety—GAD	4–40	14.8	5.03	7–32	14.9	3.74	4–40	14.7	5.71
Broad Mania	8–39	17.0	5.37	15–21	16.7	2.21	8–39	17.2	6.33
Alcohol dep	11–19	16.7	1.43	11–19	16.4	1.64	14–19	16.9	1.15
Drug abuse/dep	4–21	14.6	4.54	4–21	14.0	5.06	13–21	16.1	2.64
Suicidality	4–42	15.7	3.76	4–25	15.9	3.34	6–42	15.7	3.97
6-month ADHD	4–40	11.5	5.36	4–17	10.1	4.77	4–40	12.4	5.60*

Note: N = 739, n = raw unweighted values, % weighted values, SD = standard deviation, MDE = major depressive episode, GAD = generalised anxiety disorder, dep = dependence, Suicidality includes suicide ideation, plans or attempts, ADHD = attention deficit hyperactivity disorder.

*significant gender difference in age of onset, p < .05.

Results of logistic regression analyses examining associations between socio-demographic variables and lifetime mental health problems are presented in Table 4. Females were more likely to develop mood (*OR* = 1.565, p < .05), anxiety (*OR* = 1.609, p < .05) or any disorder (*OR* = 1.425, p < .05), compared to males. When compared to students under the age of 21, the older age group were nearly twice as likely to have experienced a major depressive episode (*OR* = 1.954, p < .01). In contrast to heterosexual students, those who said they were non-heterosexual were nearly three and a half times more likely to have a MDE, nearly four and a half times more likely to have GAD and five times more likely to have any lifetime disorder. In relation to current financial position, when compared to those who said they were poor, those who were comfortable were significantly less likely to have any lifetime disorder. In comparison to those who grew up in a rural setting, students raised in a suburban setting were more likely to have any disorder (*OR* = 1.886, p < .05).

**Table 4:** Logistic regression analyses of socio-demographic correlates of lifetime disorders.

Demographics	MDE	GAD	Any Disorder
N = 739	*OR* (95% CI)	*OR* (95% CI)	*OR* (95% CI)
**Gender**			
Female (462)	1.565* (1.1–2.3)	1.609* (1.1–2.3)	1.425* (1.0–1.9)
Male (274)	1.0	1.0	1.0
**Age**			
21 and over (178)	1.959** (1.3–2.9)	1.412 (0.9–2.2)	1.276 (0.9–1.8)
Under 21 (561)	1.0	1.0	1.0
**Sexuality**			
Non-heterosexual (66)	3.359*** (1.9–5.7)	4.411*** (2.6–7.6)	5.016*** (2.6–9.7)
Heterosexual (669)	1.0	1.0	1.0
**Current finances**			
Enough (417)	0.772 (0.4–1.5)	1.226 (0.6–2.4)	0.800 (0.4–1.5)
Comfortable (250)	0.535 (0.3–1.1)	0.653 (0.3–1.4)	0.460* (0.2–0.9)
Well to do (16)	0.608 (0.2–2.3)	0.127 (0.01–1.3)	0.396 (0.1–1.3)
Poor (56)	1.0	1.0	1.0
**Place raised**			
Large city (55)	1.186 (0.6–2.4)	1.404 (0.7–2.9)	1.574 (0.8–3.0)
Suburbs (65)	1.092 (0.6–2.1)	0.904 (0.4–1.9)	1.886* (1.0–3.4)
Small city (103)	0.619 (0.3–1.2)	1.213 (0.7–2.2)	0.963 (0.6–1.6)
Town/village (328)	0.840 (0.5–1.3)	1.012 (0.6–1.6)	1.208 (0.8–1.8)
Rural (188)	1.0	1.0	1.0

Note: MDE = major depressive episode, GAD = generalised anxiety disorder, Any disorder = Any lifetime mental health disorders or suicidality or substance problems or 6-month ADHD; OR = odds ratios, CI = confidence intervals.

Significance values

*p < .05

**p < .01

***p < .001.

Table 5 presents findings regarding treatment for emotional problems. Overall 10% of students received treatment for an emotional problem in the year prior to commencing university. Significantly more females (13.8%) received treatment than males (5.3%). A strong association was found between receipt of treatment and lifetime (χ ^2^(1, *n* = 738) = 66.454, p < .001) and 12-month disorders (χ ^2^(1, *n* = 737) = 66.142, p < .001). However, less than 20% of those with mental health problems received treatment. Moreover, 18.6% of students said that they would probably not seek help for an emotional problem. Many of these students screened positively for a range of problems. Indeed, chi-square tests for independencerevealed strong associations between probably not seeking help and both lifetime (χ ^2^(1, *n* = 733) = 7.063, p < .01) and 12-month disorders (χ ^2^(1, *n* = 735) = 5.408, p < .05), with 22.3% of those with problems saying they would not seek help. While more males (21.4%) than females (15.8%) reported that they would not seek help, the difference was not significant. The transgender and non-binary students did not get treatment in the previous year and reported they would probably not seek help.

**Table 5:** Treatment seeking, counselling and medication for emotional problems.

	Total		Male		Female		Other	
Treatment	*n*	%	*n*	%	*n*	%	*n*	χ ^2^
Psychological counselling or medication in past year	80	10.0%	16	5.3%	64	13.8%	0	13.182***
Lifetime disorder		18.6%						66.454***
No Lifetime disorder		0.3%						
12-month disorder		19.5%						66.142***
No 12-month disorder		1.3%						
Probably not seek help for an emotional problem	134	18.6%	58	21.4%	73	15.8%	3	3.454
Lifetime disorder		22.3%						7.063**
No Lifetime disorder		14.3%						
12-month disorder		22.3%						5.408*
No 12-month disorder		15.3%						

Note: N = 739, n = raw unweighted values, % weighted values, other = transgender or non-binary.

Significance values

*p < .05

**p < .01

***p < .001.

## Discussion

The current study provides baseline estimates for mental health and substance disorders, suicidality and ADHD among students commencing study at Ulster University, NI. High lifetime and 12-month prevalence rates for a range of problems were revealed. Almost a quarter of students experienced a lifetime major depressive episode, over a fifth had GAD and more than a tenth had ADHD or endorsed probable alcohol dependence. Moreover, nearly a third of students reported experiencing suicidal thoughts, plans or attempts during their lifetime. Overall, more than a half of the students experienced some lifetime disorder. Co-morbidity of problems was common with just under a fifth experiencing three or more disorders. The findings concur with other studies which found high levels of mental health and behavioural problems in the student population [3,4], although the rates revealed were higher than clinical estimates from US studies [12].

Prevalence rates for the previous 12 months were slightly lower than lifetime rates as would be expected. However, the reported rates remained high, with more than a fifth of students having a major depressive episode or GAD in the year prior to commencing university. It should be remembered that many of these students completed A levels or Leaving Certificate (ROI) exams during this time which may have impacted on their mental health and wellbeing. Additionally, since the average age of participants in this study was 21, this period for many signals their transition into adulthood which may partially account for the high rates of disorders reported. Indeed, the average age of onset for most disorders in the current study was between the ages of 14 and 17. A previous epidemiological study based on the NI population found that age of onset for many mental health problems were during these formative teenage years [40]. The authors recommended that policies and interventions should be prioritised for young people to address mental health issues before they arise.

Numerous gender differences in prevalence rates were revealed in the current study. In accordance with previous studies, females had higher lifetime rates of mood and anxiety problems [20], and suicidal behaviour. However, no significant gender differences were found for any 12-month disorders or suicidality, which contradicts previous evidence that females in this age group are at higher risk of having a current mental disorder [12]. This may reflect differences in age of onset, illness severity or duration, or help seeking, and as such merits further investigation. In line with previous studies, males were more likely to report drug abuse or dependence, although no gender variation was found for alcohol dependence contrary to other research in this area [20]. Overall females were more likely to have any lifetime disorder as well as higher rates of co-morbidity. While no significant gender difference was found for the prevalence of ADHD, age of onset was significantly lower for males.

Further analyses revealed numerous significant socio-demographic risk factors for mental health problems. In accordance with previous research, the study found that females, students over 21, those with a lower income and non-heterosexual students had the highest rates of mental health problems [5,8,23]. Students over 21 were significantly more likely to have a mood disorder. A previous study [40] found that the age of onset for mood disorders in the NI population was 32. This could suggest that many of the participants in the current study may have yet to develop a mood disorder and it is important therefore to address any issues before the onset of problems. The findings indicate that universities also need to be mindful of the increasing numbers of students from disadvantaged families with low socio-economic status. Non-heterosexual students also had a heightened risk of experiencing a range of mental health disorders and it is important therefore that information, help and support is available on campus to address their needs.

A key finding from this study, which warrants careful consideration, is the fact that many students with pre-existing problems said that they did not receive treatment and that they would not seek help for an emotional problem. This may be related to the severity of their symptoms, with some having only mild symptoms that did not require treatment. Others may not be fully aware of their problems, or they may have not been diagnosed yet. However, since many students met the criteria for a disorder it is reasonable to assume that there is unmet need and that they would benefit from treatment. Females received more treatment since they were also more likely to have a mental health problem but this is likely to only partially account for the gender difference found. Males were less likely to look for help. Some would suggest that it is due to stigma, embarrassment and an unwillingness to express emotions [25,41]. It may also be related to how serious they perceive the illness to be. One study [2] found that while many students with psychological problems were aware that they needed help, service use was low. Others propose that stigma only partially accounts for this and that universities need to look at innovative ways to encourage behaviour change [12].

It is important therefore to carefully consider strategies to encourage help-seeking behaviour, increasing the uptake of services to address these problems. This may include anti-stigma campaigns, gatekeeper training and effective screening programmes [12]. Additionally, the quality and quantity of counselling services, which are often overstretched, needs to be addressed in all universities, with flexible opening hours, prompt responses, reduced waiting lists and free sessions to students in need [42].

While studies show that the uptake of services is low in comparison to the levels of mental health problems reported in the student population, the number of students accessing counselling services has nonetheless grown in recent years. For example, a recent report found an increase of 50% in the past 5 years [43]. Increases in awareness of mental illness may have led to an upsurge in treatment seeking. Such findings are promising, indicating that more students are getting help, with early interventions and treatment helping to alleviate problems. However, a large proportion of mental health issues remain untreated. A review of the evidence suggests that it is important to have ongoing campaigns to increase awareness among students about the services and support that is available within the university and from other external sources [44].

### Limitations

While the study provides important information regarding student mental health and wellbeing, several limitations should be considered when interpreting the findings. For instance, the on-line survey utilises self-report questionnaires which are often criticised for providing inaccurate results due to recall issues. However, since many of the sample was 21 or younger, recall issues should not have a great impact. Although prevalence rates of most major mental health disorders are reported in the current study, those disorders that are not characterised easily by self-report such as psychotic disorders were not included. Another limitation of the study is that the response rate is low and hence precludes us from safely generalising to the student population within this university. Furthermore, many more females participated. Sampling weights however were applied to address these issues but possible response bias should be considered. It is difficult to determine therefore exactly how representative the current study is. Previous reports suggest that people with mental health problems may be less likely to participate in studies such as this, due to stigma or a fear that if they reveal a mental health problem that it may impact on their future career [45], which may indicate that our findings are an underestimate of the prevalence of mental health disorders. However, it is also possible that those with mental health problems may be more likely to engage in such studies due to inherent interest in the study, meaning that rates reported may be higher than the true prevalence. The rates of mental health disorders reported, however, are comparable to other studies of student mental health in the UK [9] and the general population in Northern Ireland [40].

### Conclusions

Previous studies suggest that many students have mental health and behavioural problems while at university which can impact on their wellbeing and may result in elevated attrition rates. The current study extends on these findings, providing important information on baseline rates of mental health and behavioural problems, along with help seeking in a representative sample of students commencing university in NI. Such findings mean that those in need of help are identified early and provided with information on available services. This may lead to improved retention rates and academic success, as well as maintaining or improving psychological health and wellbeing beyond the university years.

A review of the evidence suggests that it is essential to increase awareness among students about the services and support that is available, as well as providing guidance for university staff to assist students with mental health difficulties [44]. Staff should be adequately equipped to make referrals and know where students can get the help they require. Improved screening for disorders and early diagnosis are also important. By intervening early and encouraging help-seeking, mental health and behavioural problems can be treated before they escalate and grades and retention rates may be improved. The UU Student Wellbeing Survey will monitor student’s health and wellbeing throughout their time at university, by conducting longitudinal research on all those who initially consented to participate, with findings helping to inform policy makers and practice within the university setting and beyond.
